# Relationship between elevated impulsivity and cognitive declines in elderly community-dwelling individuals

**DOI:** 10.1038/s41598-020-78124-5

**Published:** 2020-12-03

**Authors:** Keisuke Sakurai, Haowei Li, Noriko Inamura, Nobutaka Masuoka, Tatsuhiro Hisatsune

**Affiliations:** 1 Department of Integrated Biosciences, The University of Tokyo, Kashiwanoha 5-1-5, Biosciences Bldg., Room 402, Kashiwa, Chiba 277-8562 Japan; 2 Community Hearth Promotion Laboratory, Mitsui Fudosan, Co., Ltd, Kashiwa, Japan; 3 Urban Design Center Kashiwanoha (UDCK), Kashiwa, Japan

**Keywords:** Psychology, Signs and symptoms

## Abstract

Impulse control disorders are recognized as one of the behavioral and psychological symptoms of dementia (BPSD). Majority of studies on the treatment of BPSD related to impulsivity have rather focused on the aggression and agitation. In particular, it has not been investigated how cognitive declines are associated with impulsivity in community-dwelling elderly people. Here, we have measured the cognitive and memory functions and impulsivity of 212 elderly community-dwelling people using a psychometric test battery and analyzed the correlation between their level of impulsivity and cognitive functions by multiple regression analysis. We found an elevation of impulsivity, which was evaluated by the Barratt Impulsiveness Scale-11, closely related to decline of cognitive functions, which were evaluated by the Montreal Cognitive Assessment and the Mini-Mental State Examination, and Logical Memory function, which were evaluated by the Wechsler Memory Scale-Delayed Recall. Then we have divided them into groups based on the severity of cognitive decline and conducted an analysis of each group, the result of which showed that as this tendency was particularly noticeable in the suspected dementia group. Therefore, we have concluded that heightened impulsivity is negatively associated with cognitive and memory functions in community-dwelling elderly people.

## Introduction

Impulsivity is a behavioral trait that could be a source of various social problems such as morbid betting, kleptomania, overeating, and violence^1–4^. Impulsivity is typically defined as a predisposition toward rapid, unplanned actions responding to internal or external stimuli without regard for the consequences they might bring to oneself or to the others^5^. Impulsivity is also recognized as an intermediate phenotype of various behavioral abnormalities, as well as a phenotype that sometimes accompanies the progression of various types of dementia, including frontotemporal dementia (FTD)^6–8^ and Alzheimer’s disease (AD)^9–12^. With the progression of dementia, cognitive functions decline in a variety of domains, and behavioral and psychological symptoms of dementia (BPSD), including impaired impulse control, are exhibited^11–14^. Worsening BPSD, makes it difficult for afflicted individuals to lead a normal social life; more often than not, people who have dementia need medical attention and caregivers’ help due to their BPSD, rather than due to a decline in memory or cognitive functions^13–15^. In AD patients, first symptoms take the form of mild cognitive impairment, in which memory loss is the main symptom. While their daily lives are still unaffected at the first stage of AD^16,17^, impulsivity may be elevated in a particular percentage (17–40%) of AD patients^18,19^.

Both clinical^20–22^ and animal studies^23–25^ have suggested the implication of serotonergic neurons at the dorsal raphe nucleus in the pathophysiology of impulsivity in the series of histochemical analyses showed the degeneration of serotonergic neurons in some of AD patients^26–31^. The accumulation of senile plaques and phosphorylated tau has been also reported to be seen in the damaged raphe nucleus in the AD brain^26,31^, and elevated impulsivity was observed in transgenic mouse models of AD^25,32–35^. More specifically, alterations in the activity of the dorsal raphe nucleus as well as the degeneration of serotonergic neurons at the dorsal raphe nucleus has been detected in an AD mouse model of APPswe/PSEN1dE9^25,35,36^. Therefore, the serotonergic system is considered to be a potential therapeutic target for AD patients^23,37,38^. On the other hand, changes in the dopaminergic system are also known to affect impulsivity^39^, and clinical evidence for influences the dopaminergic neurotransmission have on human impulsivity is provided by studies on dopamine-related changes in patients who have Parkinson's disease (PD) such as the observation of behavioral changes that meet the criteria for impulse control disorders in some of PD patients following the administration of dopamine replacement therapies^40^. It has been reported in rat studies as well that the enhanced dopamine neurotransmission increases the number of impulsive actions, which is one of key indicators of impulsivity, while those impulsive responses are suppressed by decreased dopaminergic neurotransmission^41,42^. Enhanced brain dopamine transmission also enhances delay aversion, another indicator of impulsivity, which is suppressed by a decrease in transmission of this neurotransmitter^43^. However, as impulsive actions and delay aversion are not the same type of behavioral abnormalities, the way dopaminergic system affects or triggers each negative behavioral trait may be different, and specific mechanisms of action are proposed for each behavioral symptom that suggests heightened impulsivity.

Meanwhile, several personality tests have been developed to assess the degree of impulsivity^20,44^. The Barratt Impulsiveness Scale (BIS) is one of the oldest and most widely used tests of impulsivity^44^, whereas the BIS-11 is the revised version of the original BIS by Patton et al., which includes three additional factors for assessment: motor impulsiveness, non-planning impulsiveness, and attentional impulsiveness^45^. The BIS-11 is a useful personality test that aims to measure the relationship between multidimensional impulsivity and maladaptive behavior and now translated into several languages and used worldwide^46,47^. In Japan, Kobashi and Ida have translated BIS-11 into Japanese^48^, and internal consistency, retest reliability, and construct validity of the Japanese version of the BIS-11 have been confirmed^49^.

The cognitive functions of elderly people decrease as they age^50^. However, no studies have been yet conducted on how the decline in cognitive functions are connected with high levels of impulsivity. Therefore, in this study, we aimed to investigate the relationship between cognitive decline and impulsivity using a psychometric test battery in community-dwelling elderly people.

## Results

### Participants

Participants were 212 community-dwelling individuals (83 men, 129 women) aged over 60. Age ranged from 60 to 84 years (average, 72.8 years), body mass index (BMI) ranged from 15.6 to 31.8 kg m^−1^ (average, 22.1 kg m^−1^), and years of education ranged from 9 to 21 years (average, 14.0 years) (Table 1).

**Table 1:** Participant characteristics.

Age*	72.8 ± 5.1
BMI*	22.1 ± 2.6
Years of Education*	14.0 ± 2.2
Sex (Male / Female)^#^	83 / 129

*Data are mean ± standard deviation.

^#^Data are the number of people.

BMI: body mass index; F: female; M: male.

### Multiple linear regression analyses

The skewness of the total BIS-11 scores was 0.68, while the kurtosis was 4.70, which with the test of normality performed has shown that the BIS-11 scores were not normally distributed (p < 0.001). Therefore, we have converted the BIS-11 scores to a logarithmic scale for multiple regression analysis and found the normal distribution of the BIS-11 scores (p = 0.060). A set of multiple regression analyses was performed to examine the relationship between cognitive functioning and the degree of impulsivity (Tables 2, 3 and 4). Multiple linear regression was used to examine the relation between Montreal Cognitive Assessment (MoCA) score and the BIS-11 score with several covariates. A significant regression equation that has the MoCA score as the dependable variable was found with the three explanatory variables which are as follows: (*F*_(3, 212)_ = 16.13, *p* < 0.001, *R*^2^ = 0.19). Age, sex, and the BIS-11 score were incorporated into the multiple regression model, and these three factors turned out to be the ones that showed the most significant correlations with the MoCA score (Table 2). Table 3 shows the results of a multiple linear regression that examines the relation between Mini-Mental State Examination (MMSE) score, the BIS-11 score and covariates. Here, a significant regression equation was found with the three explanatory variables (*F*_(3, 212)_ = 8.70, *p* < 0.001, *R*^*2*^ = 0.11). Among the factors incorporated into the multiple regression model, age, sex, and BIS-11 score were again significantly associated with MMSE score. Of these three factors, age and sex have shown significant linkage to the scores of both MoCA and MMSE. Table 4 shows the results of a multiple linear regression for the evaluation of the relation between WMS-DR (Wechsler Memory Scale-Delayed Recall) score, the BIS-11 score and covariates. This time, a significant regression equation was found with the five explanatory variables (*F*_(5, 212)_ = 11.45, *p* < 0.001, *R*^*2*^ = 0.22). Among the factors incorporated into the multivariable model, age, years of education and BIS-11 score were significantly associated with the WMS-DR score (Table 4). BIS-11 score was a negative predictor in MoCA, MMSE, and WMS-DR score. The correlation coefficient was 0.62 for MoCA and MMSE, 0.42 for MoCA and WMS-DR, and 0.35 for MMSE and WMS-DR.

**Table 2:** Relation between MoCA score and BIS-11 score and covariates.

	MoCA		
	*β*	*SE β*	*p*
Age	− 0.28	0.06	< 0.001
Sex	− 0.21	0.06	< 0.001
BIS	− 0.19	0.06	0.002
*R*^*2*^	0.19		
*F*	16.13***		

*β* represents a partial regression coefficient. *SE β* represents a standard error of the partial regression coefficient. *p* represents the p value of the significant test of the partial regression coefficient. *R*^*2*^ represents a coefficient of determination of multiple linear regression equations. *F* represents an f value of multiple linear regression equations. Regression equation significance, *p < 0.05, **p < 0.01, ***p < 0.001. BIS: Barratt Impulsiveness Scale-11; BMI: body mass index; MoCA: Montreal Cognitive Assessment.

**Table 3:** Relation between MMSE score and BIS-11 score and covariates.

	MMSE		
	*β*	*SE β*	*p*
Age	− 0.17	0.07	0.011
Sex	− 0.18	0.07	0.008
BIS	− 0.18	0.07	0.006
*R*^*2*^	0.11		
*F*	8.70***		

Regression equation significance, *p < 0.05, **p < 0.01, ***p < 0.001.

**Table 4:** Relation between WMS-Delayed Recall score and BIS-11 and covariates.

	WMS-DR		
	*β*	*SE β*	*p*
Age	− 0.27	0.06	< 0.001
BMI	0.12	0.06	0.066
Sex	− 0.11	0.07	0.132
Years of Education	0.29	0.07	< 0.001
BIS	− 0.14	0.06	0.033
*R*^*2*^	0.22		
*F*	11.45***		

Regression equation significance, *p < 0.05, **p < 0.01, ***p < 0.001.

### Sub-group analysis based on the score of MoCA and MMSE

We divided subjects into three groups by their cognitive test scores, with suspected dementia cut-off points of MMSE (23/24)^51^ and suspected MCI cut-off points of MoCA (25/26)^52^, and we investigated the correlation between the low test scores and the high level of impulsivity. Group 1 includes 115 participants and corresponds to the cognitive normal elderly based on two cognitive test scores (MoCA ≥ 26 and MMSE ≥ 24), while 82 participants, who are likely to be at the stage of MCI, belong to Group 2 (MoCA ≤ 25 and MMSE ≥ 24). 15 people (10 males and 5 females), highly likely to have dementia, to Group 3 (MoCA ≤ 25 and MMSE ≤ 23). No participants scored more than 26 in MoCA, or less than 23 in MMSE. As a result of analysis of covariance (ANCOVA) using the total score of BIS-11 as the objective variable, group as the fixed factor, and age, sex, and education as covariates, a significant regression equation was found (*p* = 0.004; significance test for linear regression). The remarkable difference in the total BIS-11 score between groups was quite significant (p = 0.001), and a post-hoc test revealed that Group 3 had scored much higher than the other two groups (Table 5).

**Table 5:** Sub-group analysis based on the severity of cognitive dysfunction.

	G1 (CN)	G2 (MCI)	G3 (Dementia)	*p* value
Sex (M / F)^†^	35/80	38/44	10/5*	0.006
Age^§^	71.7 ± 4.6	74.1 ± 5.2*	74.5 ± 5.5*	0.001
Years of education^§^	14.1 ± 2.2	13.8 ± 2.1	14.7 ± 2.9	0.305
BIS^¶^	59.4 ± 7.7	60.4 ± 7.6	66.9 ± 15.1*^, #^	0.001

*Significant difference from the value of G1 group, *p* < 0.05 (Bonferroni post-hoc-test).

^#^Significant difference from the value of G2 group, *p* < 0.05 (Bonferroni post-hoc-test).

^†^Data are the number of people. *p*-value for the significant difference between groups was calculated by chi-squared test.

^§^Data are mean ± standard deviation. *p*-value for the significant difference between groups was calculated by one-way analysis of variance (ANOVA).

^¶^Data are mean ± standard deviation. *p*-value for the significant difference between groups was calculated by analysis of covariance (ANCOVA).

## Discussion

This study aims to investigate the relationship between the level of impulsivity and the decline in cognitive functions in the elderly population. In the community-dwelling elderly people, we found a strong correlation between elevated impulsivity and cognitive declines. Levels of impulsivity was measured by BIS-11 questionnaire, and cognitive abilities were assessed by three psychiatric tests which are as follows: MMSE, MoCA , and WMS-DR. In this study, we found a clear negative correlation between high levels of impulsivity and cognitive functions by analyzing the scores in MoCA, MMSE, and BIS-11.

We must also discuss correlation coefficients between three cognitive test we have used. The correlation coefficient was 0.62 for MoCA and MMSE, 0.42 for MoCA and WMS-DR, and 0.35 for MMSE and WMS-DR. Guilford defines, -the magnitude of a correlation coefficient as follows:- Less than 0.20……. Slight; 0.20–0.40…….Low correlation; 0.40–0.70…….Moderate correlation; 0.70–0.90…….High correlation; 0.90–1.00…….Very high correlation^53^. Since both MMSE and MoCA were developed as a screening tests for cognitive impairment, one might expect their correlation number should be higher. However, MoCA is a more rigorous test for MCI and focuses more on language and execution functioning skills, which require higher cognitive abilities than MMSE; therefore, the correlation coefficient between MoCA and MMSE turns out to be rather moderate.

As discussed, the comparison results of MoCA and MMSE, and BIS-11 suggest a strong correlation between cognitive severity stages and the degree of impulsivity. Besides, WMS-DR is a sub-category of WMS, which is a test that evaluates the episodic memory function by recalling a meaningful story presented verbally after a while^54^. Therefore, the results regarding the relationship between BIS-11 and WMS-DR in this study might indicate relationship between impulsivity and episodic memory rather than general cognitive functions. This is the first study that focused on the negative correlation of the BIS-11 score with cognitive functions of the elderly, but the idea that the stages of dementia is closely associated with the level of impulsivity is not new; in fact, in other personality tests that assess the impulsivity-agitation domain such as Urgency Premeditation Perseverance Sensation Seeking (UPPS) Impulsive Behavior Scale^11,12^ or Cohen-Mansfield Agitation Inventory (CMAI)^9,13^, it is already shown that high levels of impulsivity are seen in some of the AD patients and it itself is one of, the symptoms of BPSD. Impulsivity-agitation domain is one of the most troublesome BPSD^13^ and measuring BIS-11 in the elderly may lead to a supplementary diagnosis with individuals with a rapid decline in cognitive functions.

We observed gender differences in cognitive decline. A multiple regression analysis of the scores of the MMSE and MoCA tests has shown that female subjects scored much higher than the male counterpart. This tendency has been already observed in MMSE test scores^55^. Although it may sound rather obvious participants’ age was also negatively associated with both cognitive and memory functions. This supports previous findings that among the clinically normal elderly, cognitive and memory functions decline with aging^56–58^.

Our study has some limitations. First, the sample size may not have been sufficient to detect all the changes in impulsivity levels that occur along with the progression of dementia. Since the sample population in this study was not patients with dementia but the community-dwelling elderly, the number of subjects with suspected dementia was relatively few. Second, since it is sometimes hard to distinguish AD symptoms from other types of cognitive impairment without a definitive diagnostic tool such as magnetic resonance imaging (MRI) scanner, which we were unable to use in this study, we cannot entirely rule out the possibility that subjects we regarded as having AD might have had other types of dementia. However, we did our best by collecting a medical history, if available, of subjects showing the signs of cognitive impairment, or by a perusal of their medical interviews with physicians to know the symptoms subjects had, before determining the type of dementia they were most likely to have. Here are the four classifications of dementia we examined: FTD, vascular dementia (VaD), and dementia with Lewy bodies (DLB). AD is the most common type of dementia, and 70% of patients with dementia is said to have AD. VaD comes next and account for 20%, whereas FTD and DLB take up only a small fraction of people with dementia—5% for each^59^. VaD comes from abnormalities formed in cerebral blood vessels and having had cerebral infarction or cerebral hemorrhage is a huge risk factor for VaD, which often takess the form of sudden dementia^60^. We have to note here that in this study, none of the subjects with suspected dementia had cerebral infarction or cerebral hemorrhage before participating in our research. On the other hand, DLB is a type of dementia caused by accumulation of Lewy bodies and characterized by a combination of cognitive decline and core symptoms that include Parkinson's syndrome, REM sleep disorder and visual hallucinations^61^, however, in none the subjects with suspected dementia were seen such characteristic symptoms of DLB. FTD is a type of dementia in which another characteristic protein which is called (Pick body) accumulate in the prefrontal cortex and temporal lobes, and its main symptoms consist of personality changes and behavioral disorders^62^. The proportion is 5% of all dementia patients, which is considerably lower than that of AD^59^. Based on these facts, we determined that the majority of the subjects with cognitive decline in this study were likely to have mild AD. The last limitation we must admit is that we did not explore other possible causes of high levels of impulsivity, mainly previously undiagnosed psychiatric disorders such as depression or adult attention deficit hyperactivity disorder (ADHD); as our focus was on the aspects and symptoms of cognitive impairment senior citizen may have, we did not conduct any test for psychiatric disorders.

Impulsivity is an intermediate phenotype that leads to various behavioral abnormalities, and impulse control disorder is recognized as one of BPSD in dementia including AD. Symptoms of BPSD vary greatly depending on the type of dementia and the severity of cognitive decline and each symptom is a subject of study on its own^63,64^. However, certain symptoms of BPSD have a tendency to develop in clusters which are called domains^65^, and they may have the same etiology, suggesting that correlated symptoms better be studied as a group^66^. Clustering approach yields following domains in BPSD: Affective, Apathy, Psychosis, Euphoria, and Hyperactivity–Impulsivity–Irritability–Disinhibition–Aggression–Agitation (HIDA) Domain^67^. As its name suggests, impulsivity belongs to the HIDA domain, which as a whole is difficult to manage and known to impose a heavy burden on caregivers^18,68^. However, as aggression and agitation are more serious problems that threaten patients’ relatives and healthcare workers, studies on the treatment of HIDA Domain usually focus on the reduction of aggression and agitation^13^, and seldom do they touch upon problems that arise from impulsive behaviors or the way to handle it. We must mention that what we call impulsivity here and we have measured using BIS-11 in this study are purely personal traits and do not have physical components in it, such as the act of aggression or agitation itself; hence it could be considered to be independent of those two traits in the HIDA Domain. What is unique about our study is that although there are studies that have focused on impulsivity as one of symptoms of dementia, none had investigated the direct connection between impulsivity and cognitive functions in general population.

In conclusion, the present study demonstrated that high levels of impulsivity are associated with poor cognitive and memory functions in the community-dwelling elderly because the elevated impulsivity measured by BIS-11 was a predictor of cognitive decline measured by MoCA, MMSE, and WMS-DR.

## Methods

### Participants

Based on the United Nations agreed cut-off for elderly age^69^, we recruited community-dwelling elderly individuals over the age of 60 who have not been diagnosed with dementia. This study was approved by the Ethics Committee of the University of Tokyo. All participants gave their written informed consent before participating in this study, which was conducted in accordance with the Declaration of Helsinki.

### Outcome measures

Cognitive functions were evaluated using the MMSE and the MoCA. Logical memory function was evaluated using the WMS-DR, which belongs to a sub-category of the WMS-Revised^70^. High scores in the MMSE and the MoCA indicate better cognitive function, and high score in the WMS-DR indicate better logical memory function. MMSE is a cognitive assessment test published in 1975 and is currently the most common screening test for dementia^51,71^. Therefore, in this study, we adopted dementia cut-off values of the MMSE for classification criteria for the group with suspected dementia. However, the diagnostic accuracy of the MMSE in detecting MCI is suggested to be rather modest^72,73^, and the MoCA was developed as a more challenging one to address this issue the MMSE had^52,74^; in fact, the MoCA is known for the improved sensitivity of detection for MCI^75,76^. For that reason, in this study, we adopted the MCI cut-off criteria of the MoCA for classification criteria for a group with suspected MCI. We divided subjects into three groups based on their cognitive scores, using suspected dementia cut-off points of MMSE (23/24)^51^ and suspected MCI cut-off points of MoCA (25/26)^52^. Group 1 (MoCA ≥ 26 and MMSE ≥ 24) corresponded to cognitive normal elderly, Group 2 (MoCA ≤ 25 and MMSE ≥ 24) corresponded to suspected MCI stage, and Group 3 (MoCA ≤ 25 and MMSE ≤ 23) corresponded to suspected dementia. Impulsivity was evaluated using the Japanese version of the BIS-11^49^. Higher scores reflect higher degrees of impulsivity.

### Statistical analysis

This study was designed assuming a Cohen's *f*^*2*^ for an effect size of 0.08 with a type 1 error protection of 0.05 two-sided and 90% of the power. The number of subjects needed was calculated to be 205. To investigate the relationship between impulsivity and cognitive/memory function, we performed a multiple linear regression analysis with MoCA, MMSE, and WMS-DR scores as objective variables, and BIS-11 score as explanatory variables. A Variance Inflation Factor (VIF) value over 2.5 is considered problematic for multicollinearity^77^. We determined that there was no multicollinearity between the explanatory variables since no factor had a VIF above 2.5. The items used as candidates for covariates were age, sex, body mass index, and years of education. Cognitive functions generally decline with age^59^, and gender differences are reportedly observed in broad areas of cognitive functions even in the elderly who do not yet show obvious symptoms of dementia^58^. It is said that years of education the elderly had also affect the scores of cognitive assessment tests. Previous studies on the association between cognitive functions and Body Mass Index (BMI) of the elderly have produced conflicting results, but as some studies have reported a positive association between them^78^, we adopted BMI as well for a candidate covariate. We calculated the skewness and kurtosis of the score of BIS-11 and tested for normality. The test of normality was performed by the Shapiro–Wilk test. As a result, the BIS-11 score in the linear scale was not normally distributed. Therefore, we converted the scores of BIS-11 to a logarithmic scale. As a result, the BIS-11 score converted to the logarithmic scale was normally distributed. In the multiple regression analyses, all variables including covariates were standardized by Z score. The combinations of candidates for covariates with the lowest Akaike Information Criterion were adopted as the final multiple regression model^79^. In the analyses of severity of cognitive dysfunction, all variables were standardized by Z score, and ANCOVA was performed with total BIS-11 score as the objective variable, the severity of cognitive dysfunction level as the fixed factor, and age, sex, and years of education as covariates. A p-value of less than 0.05 was defined as statistically significant. Microsoft Excel Add-in for multiple linear regression analysis was used as a data analytic tool in this study.

## Data Availability

Data and materials can be obtained by contacting the corresponding author.
